# Sonic Hedgehog upregulation does not enhance the survival and engraftment of stem cell-derived cardiomyocytes in infarcted hearts

**DOI:** 10.1371/journal.pone.0227780

**Published:** 2020-01-16

**Authors:** Jill J. Weyers, Jagadambika J. Gunaje, Benjamin Van Biber, Amy Martinson, Hans Reinecke, William M. Mahoney, Stephen M. Schwartz, Timothy C. Cox, Charles E. Murry

**Affiliations:** 1 Department of Pathology, Center for Cardiovascular Biology, and Institute for Stem Cell and Regenerative Medicine, University of Washington, Seattle, Washington, United States of America; 2 Center for Developmental Biology and Regenerative Medicine, Seattle Children's Research Institute, Seattle, Washington, United States of America; 3 Department of Pediatrics, University of Washington, Seattle, Washington, United States of America; 4 Department of Medicine/Cardiology, University of Washington, Seattle, Washington, United States of America; 5 Department of Bioengineering, University of Washington, Seattle, Washington, United States of America; Indiana University School of Medicine, UNITED STATES

## Abstract

The engraftment of human stem cell-derived cardiomyocytes (hSC-CMs) is a promising treatment for remuscularizing the heart wall post-infarction, but it is plagued by low survival of transplanted cells. We hypothesize that this low survival rate is due to continued ischemia within the infarct, and that increasing the vascularization of the scar will ameliorate the ischemia and improve hSC-CM survival and engraftment. An adenovirus expressing the vascular growth factor Sonic Hedgehog (Shh) was injected into the infarcted myocardium of rats immediately after ischemia/reperfusion, four days prior to hSC-CM injection. By two weeks post-cell injection, Shh treatment had successfully increased capillary density outside the scar, but not within the scar. In addition, there was no change in vessel size or percent vascular volume when compared to cell injection alone. Micro-computed tomography revealed that Shh failed to increase the number and size of larger vessels. It also had no effect on graft size or heart function when compared to cell engraftment alone. Our data suggests that, when combined with the engraftment of hSC-CMs, expression of Shh within the infarct scar and surrounding myocardium is unable to increase vascularization of the infarct scar, and it does not improve survival or function of hSC-CM grafts.

## Introduction

During a myocardial infarction (MI), cardiomyocytes within the ischemic heart wall die and, over time, are replaced by non-contractile scar tissue. Patients surviving an MI are left with a weakened heart wall and a high percentage will develop heart failure, a condition with high morbidity and mortality. The ideal treatment for MI patients would result in remuscularization of the heart wall and reestablishment of full heart function. Currently, several different approaches are being taken toward remuscularizing the heart wall, one of which is the transplantation of human stem cell-derived cardiomyocytes (hSC-CMs) into the injured heart wall post-MI.

Work by several groups has shown that hSC-CMs can be successfully transplanted into infarcted myocardium to form stable cell grafts in small [1–5] and large animal models [6–9]. These grafts establish new myocardial tissue within the injury, partially remuscularizing the infarcted heart wall and improving heart function [4–6,8,10]. Importantly, these grafts can also electrically couple with the host myocardium [7,11], thus demonstrating that a mechanical contribution from the graft is feasible, rather than simply a paracrine effect. Unfortunately, the positive effects of cell transplantation are hampered by poor cell survival [4,12].

Early efforts to transplant hSC-CMs into infarcted hearts resulted in the development of a “pro-survival cocktail” (PSC) which, combined with heat shock, inhibits multiple cell death pathways and encourages cell survival [4]. PSC enables cells to engraft, but work since then has only resulted in incremental improvements in cell survival [13], and cell death post-transplantation is still near 90% (Murry lab, unpublished observations). Low cell survival is partially overcome through the use of large doses of cells: in macaque monkeys, doses of 0.4 billion rhesus SC-CMs can achieve 16% remuscularization of the infarct scar [6], while doses as large as 1 billion hSC-CMs can remuscularize 40% of the scar [7,8]. If this relationship remains linear, full remuscularization of infarcted primate hearts would require approximately 2.5 billion cells, and equivalent doses for humans could be 7-fold larger to achieve the same effect. The requirement for such high numbers of cells makes this therapy expensive, complicates delivery and increases the risks of mutations, ploidy errors or other defects that commonly arise when growing large amounts of cells in culture, all of which may delay efforts to move this therapy toward the clinic.

One potential method of further increasing the survival of engrafted cells is to improve the environment into which they are injected. Here we proposed to reduce ischemia within the injured heart wall through increased vascularization. Indeed, higher vascularization of the heart wall is associated with lower cell death after cell injection [12]. Importantly, we and others have previously demonstrated that the coronary vasculature within an infarcted heart is capable of growing and expanding into the injury and the cell graft [14,15]. This growth response occurs at both the capillary and macrovessel levels, and occurs within the infarcted and engrafted heart wall as well as in the uninjured remote myocardium [14,15]. In the current study, we attempt to induce coronary growth prior to cell transplantation (i.e. “pre-vascularizing” the infarct), with the hypothesis that an increased blood supply within the infarct zone will reduce ischemia and provide a more hospitable environment for cell engraftment. A promising candidate to induce this “pre-vascularization” is the growth factor Sonic Hedgehog (Shh).

Shh, in addition to other developmental roles, can induce vascular growth through both canonical and non-canonical pathways whose downstream effects include the increased expression and activation of several vascular growth factors, including VEGF, angiopoietin 1 and 2, Notch, nitric oxide, and stromal cell-derived factor-1, among others [16–23]. Importantly, Shh expression leads to vascular growth in both large and small caliber vessels [16,21], and thus remodels the entire vascular tree, which, in contrast to treatments that only increase the number of capillaries, enables increases in blood flow to the tissue. In cases of ischemic injury, Shh is endogenously upregulated [21,24–27], and preventing this upregulation leads to bigger scars [26,28]. In the heart, increasing the presence of Shh post-MI through either induced expression [25], or injection of either intravenous protein [29] or Shh-expressing cells [22,30] has been reported to cause smaller scars, increased vascularization, and improved heart function. Hh signaling also has important roles in the uninjured adult heart, where it is required for maintenance of the coronary vessels [28]. Given these numerous connections to vascular growth, ischemic injury, and the coronary vasculature, Shh appears to be an ideal candidate for “pre-vascularizing” the post-MI ischemic heart wall.

In this study, we attempt to induce vascular growth within the injured heart wall via adenoviral expression of Shh prior to engraftment of therapeutic hSC-CMs, with the hopes of improving cell engraftment and overall graft function. In this environment, Shh treatment increased capillary density in the neighboring myocardium, however, it did not affect vasculature within the infarct area beyond that of treatment with cells alone. Correspondingly, the increase in coronary growth outside the infarct area was not enough to effect graft size, improve heart function, or impact scar size over that of treatment with cells alone.

## Methods

### Virus

Shh expression was induced using a human serotype 5 adenovirus (E1/E3 deleted) obtained from Vector Biolabs (Philadelphia, PA), construct Ad-h-SHH (ADV-222942), containing the human SHH gene under the control of the CMV promoter. GFP-expressing virus was cloned using the AdEasy system and the pAdTrack-CMV vector as described elsewhere [31,32]. Both viruses were amplified as in Jager et. al [33] to high titers (>2E10 virus particles (vp)/μl,) and frozen in aliquots at -80°C.

### Ischemia/reperfusion and virus injection

Animal procedures were approved by the University of Washington Animal Care and Use Committee (IACUC protocol #2225–04), and all efforts were made to minimize suffering. Surgeries for ischemia/reperfusion were performed as previously reported by our group [4,10], modified to include virus injection. Briefly, athymic male Sprague Dawley rats (*rnu-rnu*, 200–250 g, Charles River) were anesthetized, intubated, and aseptic thoracotomy was performed to expose the heart. The left coronary artery was occluded in a snare for 60 minutes, and ten minutes after reperfusion, 50 μl containing 1E8-1E10 viral particles diluted in cell culture media was injected intramyocardially into the infarct using in 3 or 4 injection sites to ensure injectate spread evenly throughout the blanched area. For blinding purposes, viral doses were prepared by a third party, and dose identity was kept hidden for the duration of the experiment.

### In vivo viral dosage tests and qRT-PCR

Viral expression of hShh was tested in infarcted rats to ensure expression surpassed the endogenous post-infarct expression [25,27]. Rat hearts were infarcted and injected with virus, as descried above. Twelve animals were enrolled in this part of the study, but only nine survived to the experimental endpoint, due to complications post-MI. We therefore ended with three animals that received GFP virus, and two animals for each dose of Shh (6 total). Hearts were harvested seven days post-MI and left ventricles were manually divided into three regions: the infarct, border zone (~2–3 mm surrounding the infarct), or remote uninjured ventricular myocardium. RNA was extracted using the E.Z.N.A. Total RNA Kit (Omega Bio-Tek) and quantitative reverse transcription PCR (qRT-PCR) was performed using standard techniques and the species-specific probes listed in Table 1. Reactions were run for 50 cycles in an ABI 7900HT RT-PCR machine. Samples were run in triplicate and results were averaged, normalized to GAPDH, and compared to levels from the GFP treated samples. Analysis was done using Excel (Microsoft). Graphs were produced in Prism (GraphPad).

**Table 1:** qRT-PCR probes.

Gene	Tag	Manufacturer	Catalog #
Rat GAPDH	FAM	Life Technologies	Rn99999916_s1
Human Shh	FAM	Life Technologies	Hs00179843_m1
Rat Gli1	FAM	Life Technologies	Rn01504244_g1
Rat Ptch1	FAM	Life Technologies	Rn0152793_m1
Rat GAPDH	SYBR	RealTimePrimers.com	VRPS-6986
GFP	SYBR	RealTimePrimers.com	CHPS-1 GFP

### Differentiation of hiPS-CMs and cell injection surgeries

All experiments were approved by the University of Washington Embryonic Stem Cell Research Oversight Committee (ESCRO). The IMR90 hiPSC line was kindly provided by WiCell Research Institute (Madison, WI). IMR90 hiPSCs were differentiated into cardiomyocytes (hSC-CMs) as in Lundy et al [34]. Cardiomyocytes resulting from the differentiation of stem cells have been extensively characterized in previous publications which confirm cardiac identity and morphology via sarcomeric structure, contractility, calcium handling, electrophysiology, and the expression of multiple markers (Nkx2.5, cardiac troponin, sarcomeric proteins, etc.) [1–4,34–38]. All cells used for this study were differentiated in the same differentiation run. Briefly, IMR90 cells were cultured in feeder-free conditions on Matrigel-coated plates (BD Biosciences) and fed with a mouse embryonic fibroblast-conditioned medium supplemented with 4ng/mL basic fibroblast growth factor. To induce cardiogenesis, cells were switched to insulin-free RPMI-B27 media (Gibco). Cultures were supplemented with recombinant human activin A on day 0 (R&D Systems) and recombinant human bone morphogenetic protein-4 on days 1–4 (R&D Systems). Cells typically began beating spontaneously on approximately day 14 postinduction. Cells were transiently heat-shocked at 42° on day 19, and cryopreserved on day 20 as in Xu et al [38]. On day 20, flow cytometry confirmed 69.9% of cells showed cardiac troponin T+ cardiomyocyte identity.

On the day of cell injection, cells were thawed and prepared for injection as in Laflamme et. al [4]. On average, the post-thaw cell viability was approximately 88% (by trypan blue uptake). Cell injection surgeries were done as previously published [4,10]. Briefly, four days after MI surgery, animals were anesthetized and thoracotomy was performed to expose the heart. 40 μl of cells in Matrigel and media (~10 million cells) were injected into the infarcted myocardium in 1–4 injection sites to ensure the cells spread throughout the infarct area. The timepoint of cell injection four days post-MI and virus injection was chosen because 4 days is an optimal stage of infarct repair for cell therapy [39], and also because it should allow sufficient time for Shh to begin to induce vascular growth; Lavine et. al showed that induced Shh expression in adult hearts leads to increased vascularization by 5 days post-induction [17]. Twelve animals were enrolled in this portion of the study, with ten surviving until the planned endpoint. The final n was: five animals received the Shh virus, and five received the GFP virus. All 10 received cells.

### Echocardiography

Heart function was monitored via echocardiography using a Vevo 2100 (VisualSonics) at three time points: before MI induction, after MI but before cells are injected, and just prior to sacrifice at the two week endpoint. Systolic and diastolic dimensions, fractional shortening, and ejection fraction were calculated using VisualSonics software and tabulated in Excel (Microsoft). Graphs were produced in Prism (GraphPad).

### Heart perfusion as preparation for μCT scans

Hearts were prepared for μCT analysis through the perfusion of Microfil (Flowtech) as previously published [40] with minor modifications. In brief, at sacrifice, rats were injected with a lethal dose of ketamine/xylazine. While their heart was still beating, their chest was opened, and 50 U of heparin (in 0.5 ml saline) was injected intravenously through the inferior vena cava, and after allowing 3 minutes for heparin to circulate, 3 ml saturated KCl was injected to arrest the heart. The descending aorta was cannulated and, using a pressurized perfusion system, hearts were retrogradely perfused at 100 mm Hg with vasodilator (4 mg/L papaverine and 1 g/L adenosine in PBS), fixative (4% paraformaldehyde), and the radiopaque contrast agent Microfil (Flowtech) mixed to the manufacturer’s specifications. After the Microfil cured, hearts were removed from the animal and fixed overnight in 4% paraformaldehyde (PFA) before being stored in 70% Ethanol. An additional three healthy control animals were used for these experiments.

### Micro-computed tomography (μCT) and 3D analysis

Scans were performed using a Skyscan 1076 (Skyscan/Bruker) μCT scanner at 9 μm spatial resolution using the following settings: 40 kV, 70 mA, no filter, 3000 ms exposure, rotation step of 0.5°, 180° scan, and 3 frame averaging. Raw scan data were reconstructed to a 3-dimensional (3D) slice dataset with an isotropic resolution of 18 μm using the software NRecon V1.6.1.0 (Skyscan/Bruker), and analyzed using CTan (Skyscan/Bruker), and Analyze 10.0 (Analyze Direct) as previously described [14]. Branching analysis was performed using the branching analysis add-on in FIJI (ImageJ, NIH). Vascular parameters were measured in separate 3D datasets for each vascular network. Vessel size and density were calculated using 2D slices from the entire volume, while percent vascular volume was calculated as a percentage of the 3D volume of soft tissue (i.e. heart tissue).

### Histology

Tissue was prepped for histology via standard paraformaldehyde-fixed paraffin-embedding techniques. Paraffin blocks were stored at 4° to enhance the extraction of mRNA for qRT-PCR of embedded tissue (see S1 Methods). Microtome sections were cut at 4 μm thickness. Immunohistochemistry was done using standard techniques with the antibodies, lectins, or stains listed in Table 2. Antibody labeling was detected using avidin-biotin amplification kits (ABC Kit, Vector Labs) with either horseradish peroxidase and the ImmPACT NovaRED chromogen (Vector Labs), or alkaline phosphatase with the BCIP/NBT chromogen (Vector Labs). Slides were then dehydrated and coverslipped in permount (Fisher).

**Table 2:** Antibody list.

Antigen	Antibody Type	Dilution	Clone/Catalog #	Manufacturer/Source
β-myosin heavy chain	Mouse monoclonal, unconjugated	1:10	A4.951	Developmental Studies Hybridoma Bank, provided by HM Blau [41]
Griffonia Simplicifolia Lectin I (Isolectin B4)	Lectin, Biotinylated	1:200	B1205	Vector Labs
Smooth Muscle Myosin Heavy Chain	Rat Monoclonal, Unconjugated	1:1000	KM3669	Kamiya
Goat anti-rat	Secondary, Alkaline Phosphatase Conjugated	1:500	112-055-003	Jackson Immunoresearch
Goat anti-mouse	Secondary, Biotinylated	1:500	115-065-003	Jackson Immunoresearch

### Histological quantification

Grafts were detected using an antibody for β-myosin heavy chain [42], a marker that is strongly expressed in the immature human cardiomyocytes of the graft, but weakly expressed in adult rat myocardium [1]. To determine the total number of cells that make up each graft in three dimensions, nuclei within β-myosin heavy chain-positive cells were counted using Sedeen Viewer histology software (Pathcore). The average 2D cell density and area per graft cell was calculated and converted to cell volume, assuming isotropic cell shape and density throughout. Total 2D graft area was determined using histological sections taken 1 mm apart using Photoshop (Adobe) and converted to a 3D volume, integrating at 0.1 mm slice thickness. Total 3D graft cell numbers were extrapolated by dividing graft volume by cell volume. Scar size was calculated in 2D using a picrosirius red stain (Direct Red 80, Sigma, 365548) to detect collagen, with a fast-green (Fast Green FCF, Sigma, F7258) counterstain. Collagen content was quantified in Photoshop (Adobe) via color-specific pixel counting of the LV.

Vessels were detected using the Isolectin B4 lectin, which labels endothelial cells [43,44]. Digital frames were randomly chosen from higher resolution views, but care was taken to avoid regions of myocardium where the capillaries ran parallel to the tissue section so as to ensure more accurate counts of vessel density and cross-sectional size. This lectin is also known to cross-react with immune cells such as macrophages, so vessels were manually identified to avoid including non-vascular staining. Final vessel quantifications were performed with custom Matlab (Mathworks) scripts.

### Statistical methods

For all analyses, data was compiled in Matlab or Excel (Microsoft), and statistics were calculated in Prism (Graph Pad Software). Comparison between two groups was done via Student’s T-test. Comparison between groups of more than 2 were done using ANOVA with post-tests as follows. For viral dosing analysis, ANOVA compared data within the same region (i.e. the four doses), followed by a Dunnet’s multiple-comparison post-test to compare each Shh dose to the control GFP dose from the same region. Significance is reported as multiplicity-adjusted P values, which account for the size of the original group being compared [45]. For μCT analyses, ANOVA was followed by Tukey’s multiple-comparison post-test to compare each group to each other. Significance is reported as P values. For all analyses, a P value <0.5 was considered statistically significant.

## Results

### Shh-expressing adenovirus injected into infarcted rat myocardium results in dose-dependent upregulation of Shh and its downstream pathway components

**Fig 1:**
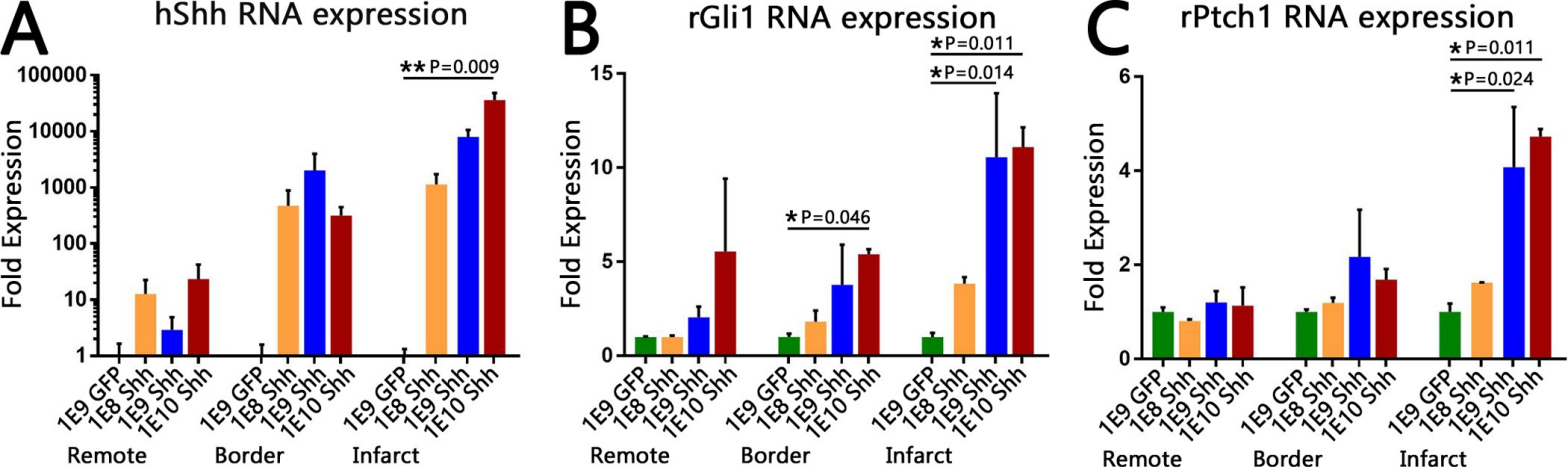
Injection of hShh virus increases expression of hShh RNA and induces Hh pathway activation within infarcted myocardium. Fold expression levels of the indicated RNA within the remote uninfarcted LV, the border zone, and the infarct after injection with either 10^9^ vp of the GFP virus, or the indicated dose of Shh virus. Error bars are SEM. n = 3 animals for GFP injection, n = 2 animals for each Shh dose. (A) Human Shh RNA expression levels, depicted on a logarithmic scale. Expression within the infarct was dose-dependent, with the highest dose inducing over 10,000 fold higher expression of hShh RNA than the GFP virus in the same region. (B, C) Rat Ptch1 (B) and rat Gli1 (C) RNA expression levels, depicted on linear scales. Both genes showed statistically significant increases in RNA expression within the infarct for the two highest doses of Shh virus.

To determine an appropriate dose of the Shh virus, we injected three different doses of Shh virus into the infarcted area of rat hearts: 1x10^8^, 1x10^9^, 1x10^10^ vp of Shh virus, or 1x10^9^ vp of GFP virus, doses determined based on work by Kass-Eisler et. al [46]. Seven days post-virus injection, qRT-PCR revealed that all three Shh viral doses resulted in an increase in hShh RNA over that of GFP virus controls in each region of the heart (infarct, border, and remote uninfarcted myocardium; Fig 1A). This increase was strongly dose-dependent in the infarct region, where the lowest dose of virus induced an approximately 1000-fold increase in hShh RNA, and each increasing dose led to an additional several thousand-fold increase in expression (Fig 1A). Similar upregulation was seen in animals that received both virus and cells (S1 Fig), confirming that injection of hSC-CMs does not interfere with adenovirus-mediated upregulation of Shh.

To determine if viral upregulation of human Shh RNA expression led to activation of the Hh pathway, we performed qRT-PCR on Ptch1 and Gli1, pathway components that are upregulated upon pathway activation [47–49]. In the infarct zone, the two highest doses of Shh virus both upregulated Gli1 around 11-fold (1E9 dose: 10.5 fold, 1E10: 11.1 fold, Fig 1B) and Ptch1 approximately 4.5-fold (1E9: 4.1 fold, 1E10: 4.7 fold, Fig 1C). The lowest dose of virus gave a more muted increase in Gli1 (~4-fold; Fig 1B) and negligible increase in Ptch1 (Fig 1C). Outside the infarct zone, a moderate increase in Gli1 RNA was seen in the border zone (up to approximately 5-fold higher; Fig 1B), but no other region showed significant increases of either gene (Fig 1B and 1C). Thus, pathway activation was limited to the region of highest hShh expression. Overall, these data confirm that the virally-expressed hShh RNA produces functional Shh protein that is indeed capable of activating the canonical Shh pathway. Since the two highest doses (1E9 and 1E10 viral particles) resulted in similar levels of pathway activation, we continued with the lower of the two doses for the remainder of our experiments, so as to avoid any potential viral cytotoxicity.

### Shh expression in infarcted and cell engrafted hearts does not enhance cell engraftment to improve heart function or decrease scar size

**Fig 2:**
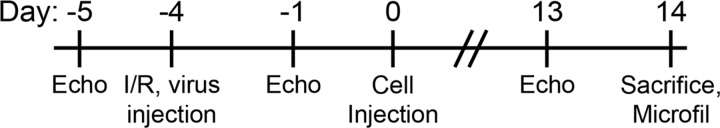
Experimental design. Time points are shown for MI, virus injection, cell injection, and endpoint measurements.

**Fig 3:**
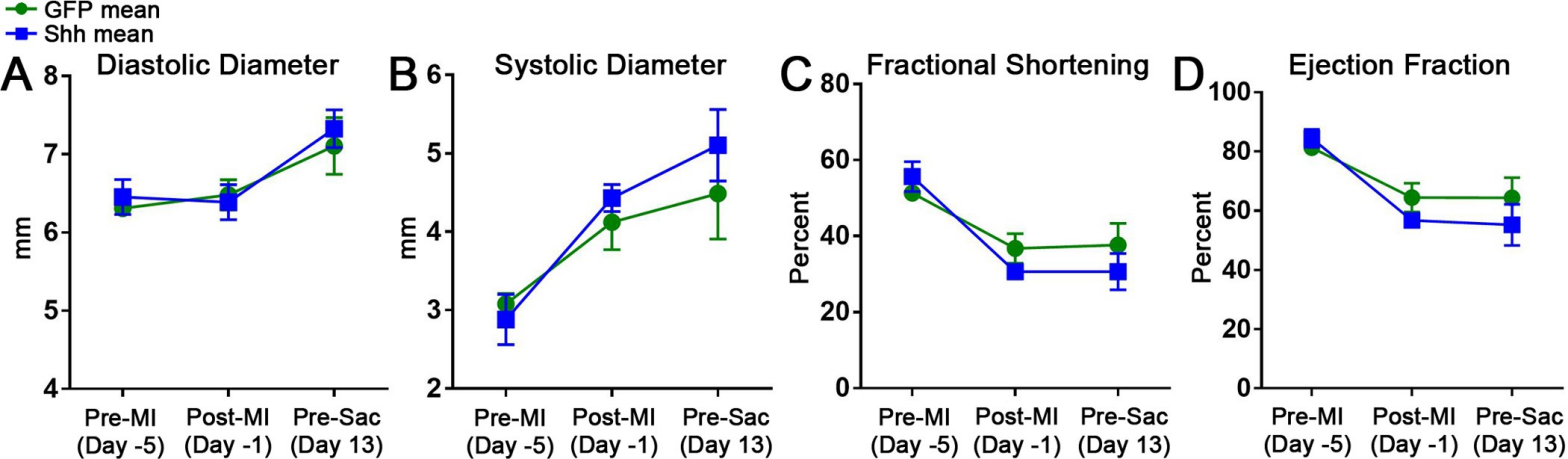
Echocardiographic measurements of heart function show no difference between cell engraftment alone versus with Shh. Short axis measurements of diastolic diameter (A), systolic diameter (B), fractional shortening (C), and ejection fraction (D) at the indicated time points. Error bars are SEM. Long axis measurements also showed no change between GFP and Shh treatments (data not shown).

**Fig 4:**
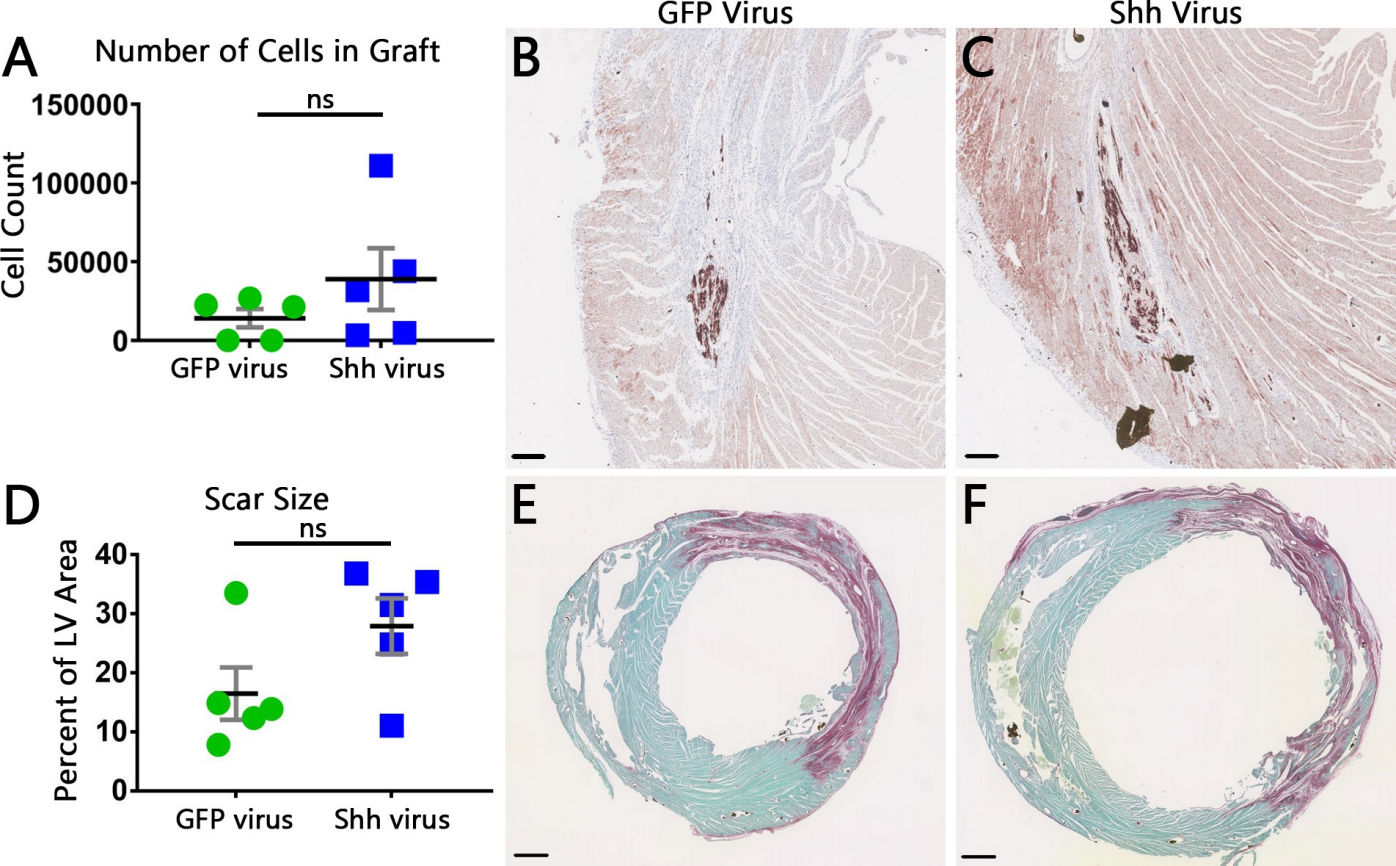
Histological measurements of graft and scar size show no significant change with Shh treatment. (A) Graph showing the total number of cells that makeup the graft for each heart. Black line is mean, error bars are SEM. (B,C) Representative images of hSC-CM grafts within the infarct scar of GFP-treated (B) or Shh-treated (C) hearts, visualized immunohistochemically via an antibody against β-myosin heavy chain (brown). Scale bars = 0.2 mm. (D) Quantification of scar size for each heart. Black line is mean, error bars are SEM. (E,F) Representative images of typical infarct scars stained with picrosirius red in hearts from GFP-treated (E) or Shh-treated (F) rats. Scale bars = 1 mm.

We next determined if increased Shh expression in infarcted hearts that were engrafted with therapeutic hSC-CMs could improve graft size and function over that of cell engraftment alone. Rats underwent MI surgery and virus injection, followed by cell injection four days later, as depicted in Fig 2. Echocardiographic measurements of systolic and diastolic diameter, fractional shortening, and ejection fraction taken before MI, 3 days post-MI, and 13 days post-cell injection, all showed no differences between animals treated with the GFP virus versus those treated with Shh virus (Fig 3). Fourteen days post-cell injection, animals were sacrificed and hearts were processed for histological evaluation of the scar and graft. A look at hSC-CM grafts revealed no change in either the density of graft cells (2747 ± 512 vs 3166 ± 1130 cells per sq mm for GFP virus and Shh virus, respectively) or average cell size (19.33 ± 1.968 μm diameter in GFP treated hearts, vs 18.66 ± 3.656 μm diameter in Shh treated hearts) between groups. Calculation of the total number of cells surviving to form each graft also showed no significant change (14,115 ± 13,045 for GFP treated vs 38,954 ± 43,845 for Shh treated rats; Fig 4A–4C). Histomorphometric measurements of scar size also revealed no significant difference between groups (16.49 ± 9.887% of LV area in GFP treated vs 27.91 ± 10.51% of LV area in Shh treated groups; Fig 4D–4F). Thus, activation of the Hh pathway within the infarct region does not increase graft size, and therefore did not improve heart function or scar size beyond that of treatment with cells alone.

### Shh increases vascular density within the remote ventricle, but not within the infarct scar

Our initial results indicated that increased Shh expression within infarcted hearts prior to cell engraftment had failed to improve heart function or increase graft size. However, it was still unclear if Shh treatment failed because it did not induce vascular growth, or because the induced vascular growth failed to have an effect. Therefore, we investigated further to determine if Shh expression had induced the expected vascular growth.

**Fig 5:**
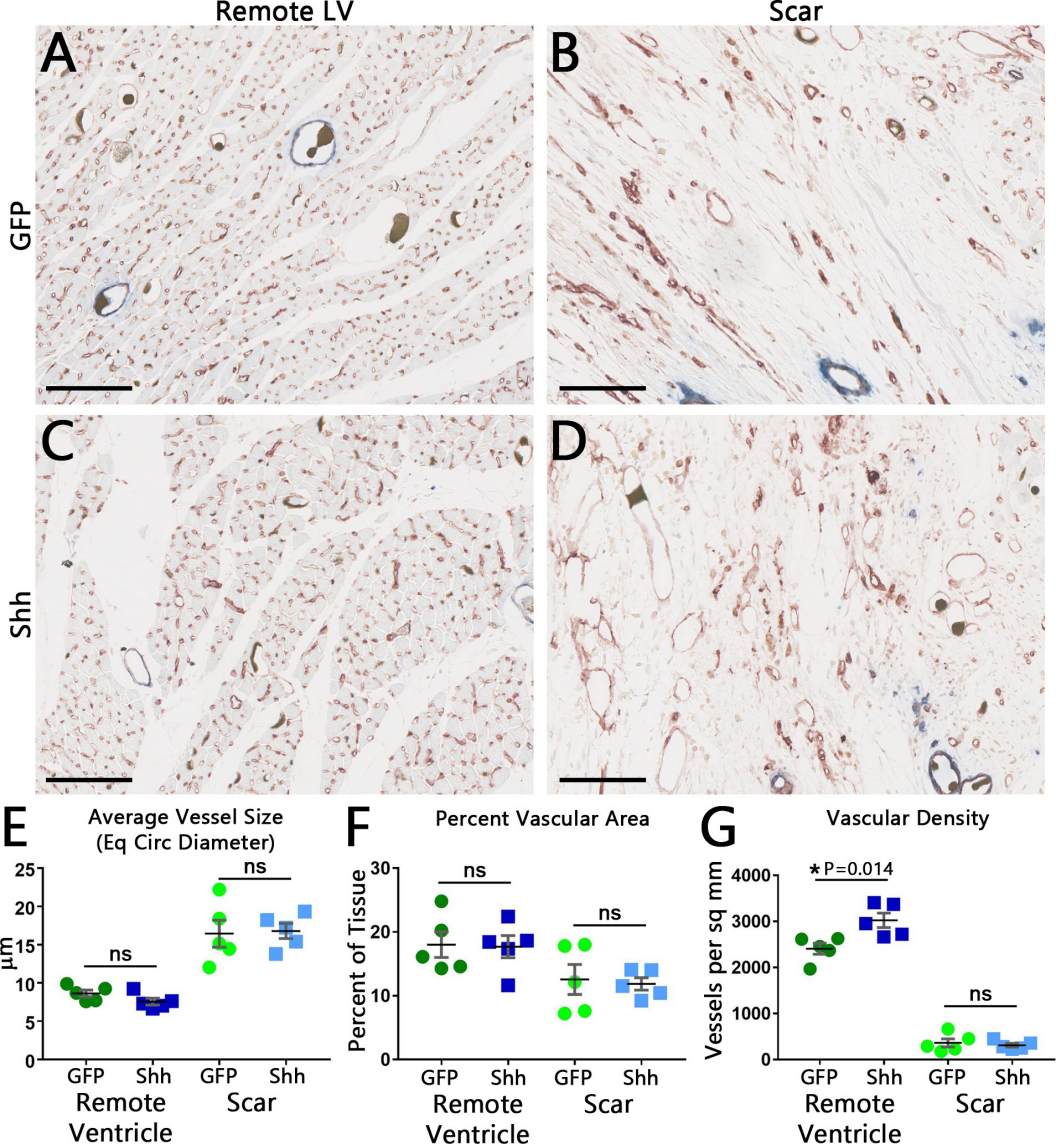
Histology of coronary vasculature reveals a Shh-induced increase in vascular density outside the infarct area. (A-D) Representative frames of the remote left ventricle (A,C) or infarct scar (B,D). Vessels were identified using the endothelial cell marker Isolectin B4 (brown, blue staining shows a smooth muscle cell marker, smooth muscle myosin heavy chain, not quantified in this figure). Although Microfil often contracts and falls away during processing and staining, some vessels have retained it (dark, granular blobs in vessel lumens). Bar = 100 μm. (E-G) Quantification of vascular parameters shows no difference between GFP-treated and Shh-treated hearts for vessel size (E) and percent vascular area (F). Vascular density, however, is increased in the remote myocardium, but not within the scar (G). Black line is mean, error bars are SEM.

We first determined if there was a quantifiable difference in the vasculature using histology. Histological analysis using the endothelial cell marker Isolectin B4 showed no obvious visible differences in the vasculature between GFP treated or Shh treated hearts (Fig 5A–5D). However, quantification revealed that vascular density increased in hearts treated with Shh, but only in the remote ventricle; the scar region showed no change (Fig 5G). Other vascular parameters, including average vessel size, percent vascular area (Fig 5E and 5F), average major and minor axis lengths, average orientation of the major axis, and average eccentricity for vessels (data not shown), all showed no difference between Shh treatment and cells alone. Thus, activation of the Hh pathway in the scar does induce an increase in the number of vessels present within heart tissue outside the scar, but has no effect within the injury where revascularization and successful cell engraftment are most needed.

### Shh expression does not affect larger vessels or the coronary branching pattern beyond that of cell injection alone

**Fig 6:**
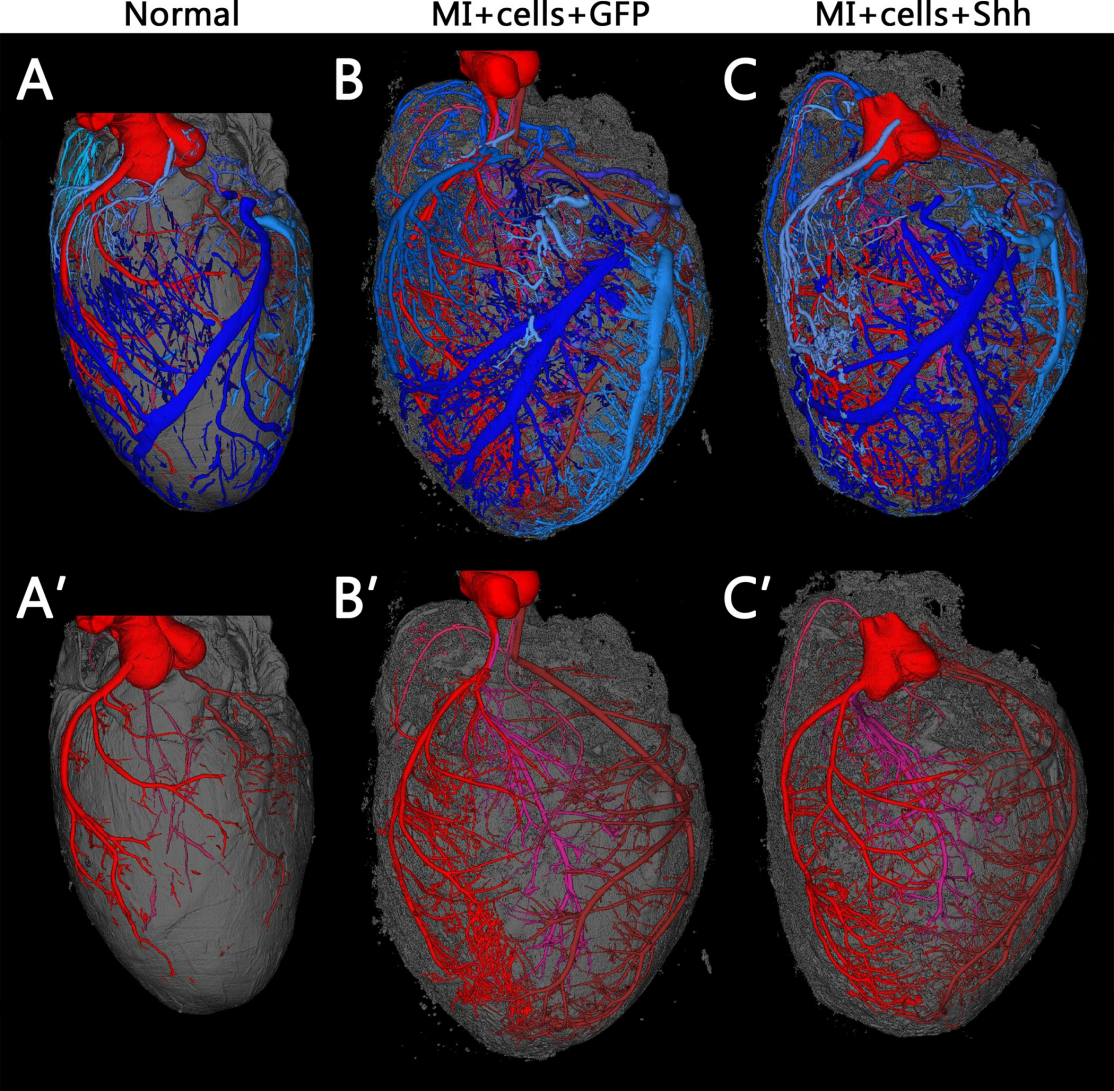
Coronary vascular trees are not visibly different between Shh and GFP treated hearts post-cell engraftment. Typical 3D reconstructions of coronary vasculature in normal uninfarcted hearts (A) versus hearts that were infarcted and injected with hSC-CMS after treatment with either GFP virus (B) or Shh virus (C). Vessels are segmented and false-colored such that arterial networks are shades of red, venous are blue. Myocardial tissue is shown in gray. The top panels show all vessels and are also available as S1–S3 Videos. Bottom panels show only arterial networks. Both groups of infarcted hearts show hypertrophy and an increase in vasculature visible by μCT, but there is no obvious visible difference between hearts treated with GFP or Shh.

Histological analysis of the vasculature provides information mostly centered on capillaries and smaller vessels easily observed in histological sections. Therefore, to focus on the effect Shh expression may have on larger vessels and on the coronary vascular tree as a whole, we performed micro-computed tomography (μCT) on cell-engrafted and virus injected hearts, in addition to normal, uninfarcted untreated controls. The resulting 3D reconstructions show that, in conjunction with some post-MI hypertrophy, both the GFP and Shh treated hearts exhibit a vast increase in vasculature from that of untreated hearts. However, there is no visibly discernable difference between the GFP and Shh treated hearts alone (Fig 6 and S1–S3 Videos).

**Fig 7:**
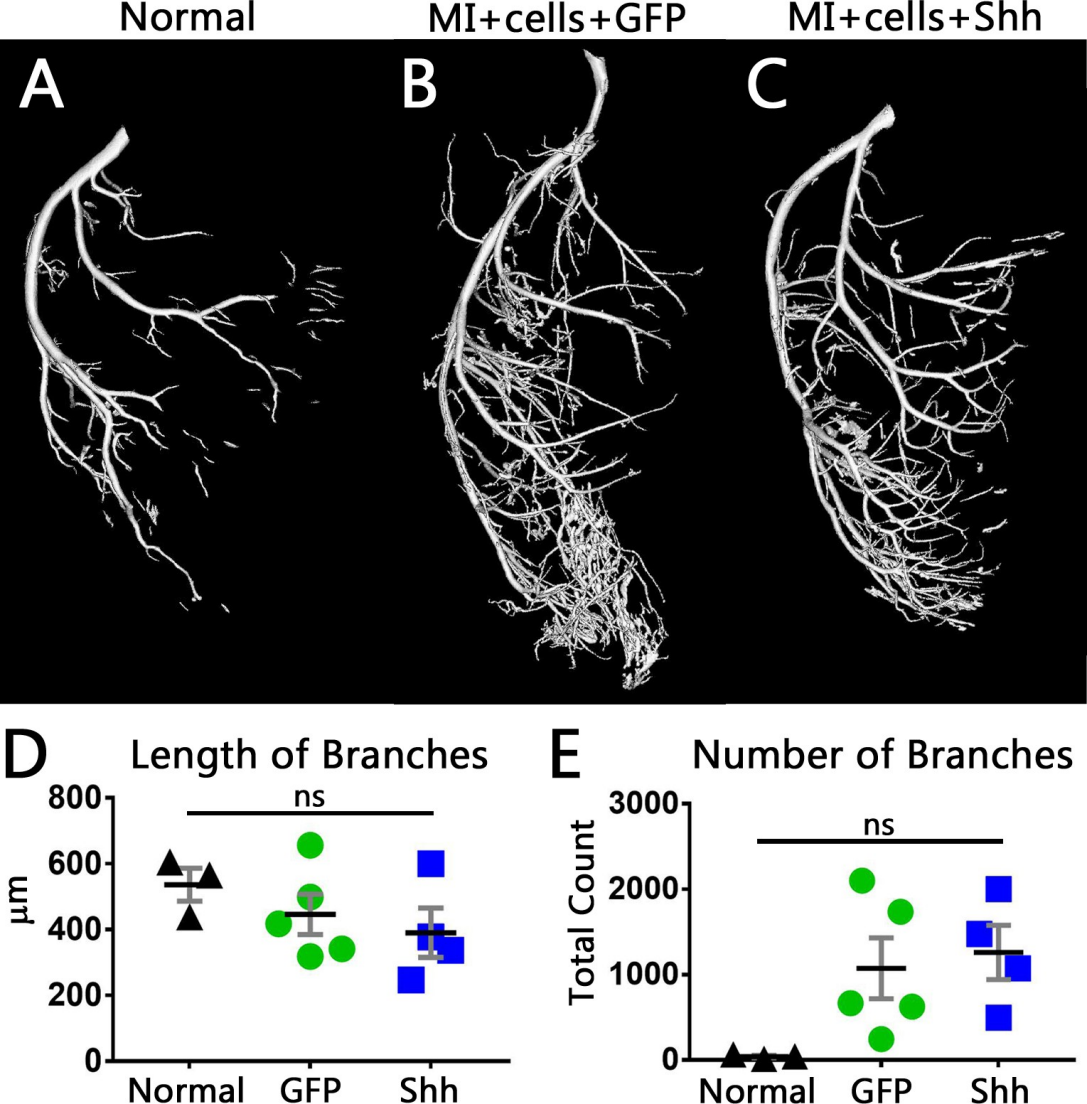
Measurements of branches within the LCA tree shows a trend toward shorter branches and more branches in the Shh treated hearts. (A-C) 3D reconstructions of the LCA within normal hearts (A) or hearts that were infarcted, and injected with hSC-CMS after treatment with either GFP virus (B) or Shh virus (C). (D,E) Quantification of the LCA branching structure. Shh treated hearts trend toward shorter branches (D) and more branches (E). Black line is mean, error bars are SEM.

To determine if there was any measurable difference in the post-MI vascular growth, we quantified the branching structures of each major arterial network. Using skeletonizations of the left coronary artery (LCA) and right coronary artery (RCA) trees from each heart, we determined the average number and length of branches present. Vessels within the LCA trended toward shorter branch lengths in both GFP and Shh treated hearts (Fig 7D), an effect which was slightly more evident with Shh treatment than GFP expressing virus. Both experimental groups also showed the corresponding trend of an increase in the number of branches as compared to normal controls, though numbers varied widely (Fig 7E). Calculations of tortuosity, however, showed no difference between Shh and GFP treatments (not shown). Measurements within the RCA also showed no change in branch length (Fig D in S2 Fig) or tortuosity (not shown), but both experimental groups trended toward an equivalent increase in the number of branches (Fig E in S2 Fig). While these trends are intriguing, none of these results reached statistical significance.

**Fig 8:**
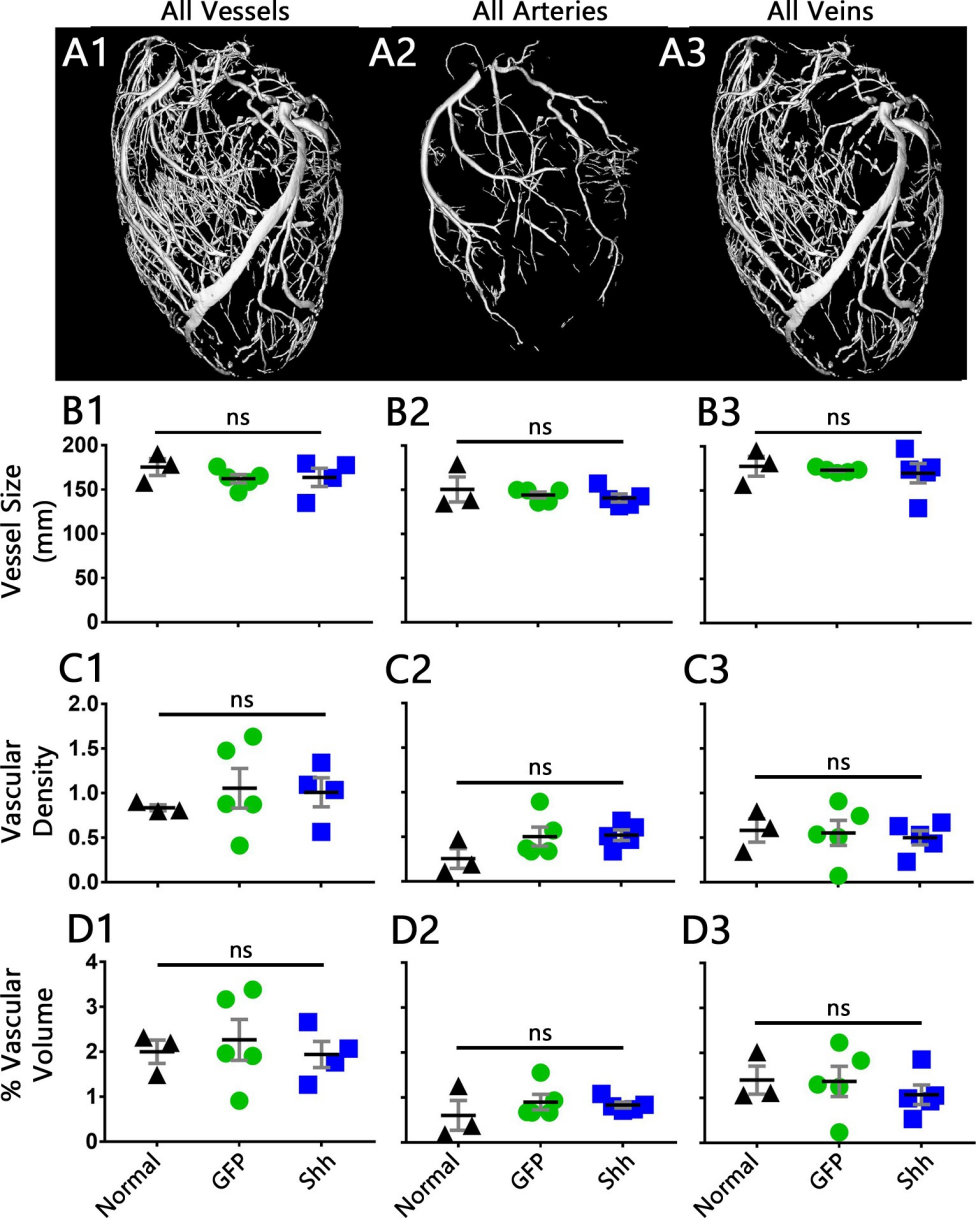
Analysis of vascular measurements shows no change between Shh and GFP treated hearts. (A1–3) 3D reconstructions of the vasculature within a normal heart, segmented to show the vascular subset analyzed within each column: all vessels (column 1), all arteries (column 2), all veins (column 3). Datasets for LCA, RCA, and LCV are in S3 Fig. (B-D) Quantification of vascular measurements, as specified. Vessel size is equivalent circular diameter (2D vessel cross sections, B), vascular density is number of vessels per square mm (2D cross sections, C), and percent vascular volume is the 3D volume of vessels compared to the 3D volume of heart tissue (D). Black line is mean, error bars are SEM.

We further quantified the larger vasculature by comparing vascular parameters within datasets consisting of all vessels, all arteries, all veins, the LCA, RCA, or left coronary vein (LCV). The average vessel size in each of these groups showed no change between normal, GFP-treated, or Shh-treated hearts (Fig 8B and Fig B in S3 Fig). Measurements of vascular density and percent vascular volume, however, both revealed a significant difference within the RCA (Fig C2 and D2 in S3 Fig), but post-tests show that this difference is between the normal controls and the infarcted groups, not between the Shh and GFP treated samples. No other measurements showed any significant change between any of the groups (Fig 8 and S3 Fig). Therefore, while infarction and human cardiomyocyte transplantation induce remodeling of the coronary vasculature, treatment with Shh does not affect the size or density of the larger vasculature; Shh expression fails to induce changes within the larger coronaries post-MI beyond that of cell injection alone.

## Discussion

In this study, we tested the hypothesis that increasing Shh expression within the infarcted heart wall would “pre-vascularize” the injury, and in turn, improve engraftment of hSC-CMs and the overall effect of cell-based therapy. Adenovirus-mediated overexpression of Shh in the infarcted and cell engrafted heart wall failed to improve heart function, graft size and infarct size beyond that of cell injection alone. Further investigation revealed that this failure was due to a muted vascular growth response. Shh treatment successfully increased intramyocardial vascular density, but only in myocardium outside the infarct zone, and only at the capillary level; capillaries within the scar and engraftment area, as well as large vessels and the vascular tree as a whole were not affected.

This study attempted to combine two previously validated therapies: cell injection, and Shh-induced vascular growth. Successful engraftment of hSC-CMs post-MI has been shown in several previous publications, where it improves cardiac function and remuscularizes the injured area [4,6,8,10,11]. Grafts also reduce scar size [6–8], and induce vascular growth [7,14]. Expression of Shh is reported to increase myocardial vascular density, decrease tissue fibrosis, and improve heart function post-MI [22,25,27,29,30,50]. We had hoped these therapies would combine to further improve the efficacy of cell engraftment: Shh would “pre-vascularize” the injured heart wall to first, make it more hospitable for injected hSC-CMs, and second, vascularize the surviving graft faster to encourage its growth and maturation. Together, this would lead to bigger, more effective grafts, and result in synergistic improvements of heart function. Unfortunately, this did not occur, as the expected vascular growth was instead a minimal increase of only capillaries, and in the wrong location to support the injected cells.

Our failure to induce vascular growth within the scar post MI is in opposition to several studies that report Shh-induced post-MI vascular growth within the infarct scar [22,25,30,50], however, there are several technical differences between these studies and ours. First, our study was done in rat, but the majority of these studies used mouse. Second, our study induced MI via an ischemia-reperfusion model, while the others used permanent ligation. These two MI methods result in slightly different scars and lead to slightly different repair mechanisms which may change how the tissue responds to Hh signaling. We note, however, that most patients are reperfused, and hence, this better models human disease. And third, we are the only study that upregulated Shh via adenovirus; other studies injected naked DNA constructs [25,50] or cells that were engineered to express Shh [22,30]. While we would expect that the method of Shh delivery would not matter, perhaps there were synergistic effects between Shh and the other’s delivery mechanisms that somehow enhanced the effect of Shh in those contexts beyond what we were able to achieve with viral delivery. The differing methods of upregulation also result in vastly different levels of increased Shh expression, which may result in different cellular responses. In the developing retina, low Shh concentrations induce axon growth from retinal ganglion cells, whereas high concentrations inhibit growth [51]. It is unknown if there is a similar concentration effect influencing vascular growth in the heart, but this does introduce the possibility that Shh expression may need to be within a precise range to stimulate vascular growth. However, studies reporting Shh-induced vascular growth post-MI had a wide range of Shh mRNA upregulation, from 12-fold [22] to as high as 20,000-fold [30]. Therefore our expression increase of 10,000-fold is still in line with what others have reported to be effective.

While our data failed to reproduce the post-MI Shh-induced vascular growth seen elsewhere, we did see an increase in capillary density outside the scar, indicating that Shh treatment did have some effect. Interestingly, Ahmed et al also found that adding Shh to their MSC-based post-MI treatment failed to induce vascular growth within the scar, but did result in an increase outside of the infarct area [22]. Importantly, while the normal adult heart wall has several cell types that can respond to Shh [28,52], other groups have found that Shh signaling must be transduced through the cardiomyocytes in order to induce angiogenesis post-MI [27] and to maintain myocardial capillaries in healthy hearts [28]. Interestingly, a similar relationship exists between angiogenesis and skeletal myocytes, where the Hh-induced gene Gli3 must be expressed specifically within skeletal myocytes to induce angiogenic growth within skeletal muscle post-ischemia [53]. Therefore, inducing vascular growth within the scar with Shh post-MI may not be feasible, since the scar, by definition, contains few cardiomyocytes.

Our study did have some limitations. For example, it is possible that different timings of virus injection and cell injection would be more effective. While Lavine et. al. demonstrated increased vessel density in non-infarcted hearts as early as 5 days after inducing Shh-expression in cardiomyocytes [17], it is possible that this time-frame is altered within an infarct environment. A longer time in between virus injection and cell injection may allow for a more complete vascular network to form, but this is outside the scope of this study. Similarly, the timing of Shh induction in relation to infarction may influence the effectiveness of the downstream response; it may be more successful if the Shh virus is delivered a few days after the infarct, rather than at the onset when necrosis is at its highest [39]. It is also possible, though not clinically feasible, that Shh expression prior to infarct (while cardiomyocytes are still abundant), would increase vessel density, protect against infarction, and improve hSC-CM survival post-engraftment.

Given our results, additional studies need to be done to more fully understand the function of Shh post-MI. For example, it is still unclear to what extent the infarct region is capable of responding to Shh for the induction of vascular growth. We also do not fully understand the mechanisms and timeframe behind endogenous capillary growth within the scar post MI. While cell injection is still a promising therapy, if we want to improve the efficiency of cell engraftment through pre-vascularization of the infarct, we need a better understanding of what controls vascular growth post-MI, and must ensure that whatever vascular growth factor is tested induces a strong vascular growth response within the specific environment in question.

Overall, we conclude that adenoviral upregulation of Shh is an ineffective method of inducing vascular growth within the injured heart wall post-MI; it fails as a pre-treatment to improve therapeutic cell injection strategies.

## Supporting information

S1 Methods Confirmation of Shh expression and pathway activation in cell engrafted rats. (DOCX)

S1 Fig qRT-PCR results confirming Shh expression and pathway activation in cell engrafted rats. qRT-PCR results from infarcted and cell engrafted animals that received a 10^9^ vp dose of either the GFP virus or the hShh virus. RNA was isolated from tissue that had been fixed and paraffin embedded, and therefore reflects levels from within the entire heart, not the localized areas as in Fig 1. As such, these results show lower and more inconsistent upregulation. (A) hShh RNA levels were increased in all animals that received the Shh virus. (B,C) Upregulation of rPtch1 (B) and rGli1 (C) did not reach significance, but did show strong trends toward increased expression. (D,E) We also determined if hSC-CMs responded to Shh by upregulating the human forms of the downstream Hh pathway components Ptch1 and Gli1. The injected hSC-CMs do not show a measurable increase in the amounts of hPtch1 (D) or hGli1 (E) RNA as compared to those treated with GFP virus. Therefore, the engrafted cells do not contribute to the spread of pathway activation within the scar.
(TIF)

S2 Fig Measurement of branches within the RCA shows no change between Shh and GFP treated hearts. (A-C) 3D reconstructions of the RCA within normal hearts (A) or hearts that were infarcted, and injected with hSC-CMS after treatment with either GFP virus (B) or Shh virus (C). (D,E) Quantification of the branching structure. Black line is mean, error bars are SEM.
(TIF)

S3 Fig Analysis of vascular measurements shows no change between Shh and GFP treated hearts. (A1–3) 3D reconstructions of the vasculature within a normal heart, segmented to show the vascular subset analyzed within each column: LCA (column 1), RCA (column 2), LCV (column 3). (B-D) Quantification of vascular measurements, as specified. Vessel size is equivalent circular diameter (2D vessel cross sections, B), vascular density is number of vessels per square mm (2D cross sections, C), and percent vascular volume is the volume of vessels compared to the volume of heart tissue (calculations in 3D, D). Black line is mean, error bars are SEM. Reported P values are from the ANOVA analysis, not the Tukey’s post-test.
(TIF)

S1 Video 3D reconstruction of the coronary vasculature in a normal rat heart. Vessels are segmented and false-colored such that arterial networks are shades of red, venous are blue. Myocardial tissue is gray.
(MP4)

S2 Video 3D reconstruction of the coronary vasculature in an infarcted rat heart treated with hSC-CMS and GFP-expressing virus. Vessels are segmented and false-colored such that arterial networks are shades of red, venous are blue. Myocardial tissue is gray. Comparing this video with S3 Video reveals no visible difference in the post-MI vascular response with Shh treatment.
(MP4)

S3 Video 3D reconstruction of the coronary vasculature in an infarcted rat heart treated with hSC-CMS and Shh-expressing virus. Vessels are segmented and false-colored such that arterial networks are shades of red, venous are blue. Myocardial tissue is gray. Comparing this video with S2 Video reveals no visible difference in the post-MI vascular response with Shh treatment.
(MP4)
