# Supplementary material for: Sonic Hedgehog upregulation does not enhance the survival and engraftment of stem cell-derived cardiomyocytes in infarcted hearts
Source: PLoS One. 2020 Jan 16;15(1):e0227780. doi: 10.1371/journal.pone.0227780 (PMC6964843; doi:10.1371/journal.pone.0227780)
Supplement: S1 Methods — (DOCX) [file pone.0227780.s001.docx]

## Supporting Methods

### Confirmation of Shh expression and pathway activation in cell engrafted rats.

RNA was extracted from paraffin embedded tissue leftover from histological analysis using the RecoverAll Total Nucleic Acid Isolation kit (Ambion). These results represent expression levels 14 days after cell injection (19 days after MI and virus injection). qRT-PCR was performed as before, with probes in Table 1 and Table S1 in S1 Methods, in an ABI 7900HT RT-PCR machine using standard protocols. Samples were run in triplicate and results were averaged, normalized to GAPDH, and compared to the averaged levels from the GFP treated samples.

**S1 Table. Additional qRT-PCR probes**

| **Gene** | **Tag** | **Manufacturer** |
| --- | --- | --- |
| Human Shh | SYBR | Life Technologies |
| Human Gli1 | SYBR | Life Technologies |
| Human Ptch1 | SYBR | Life Technologies |
