# Supplementary figures and images for: Sonic Hedgehog upregulation does not enhance the survival and engraftment of stem cell-derived cardiomyocytes in infarcted hearts
Source: PLoS One. 2020 Jan 16;15(1):e0227780. doi: 10.1371/journal.pone.0227780 (PMC6964843; doi:10.1371/journal.pone.0227780)

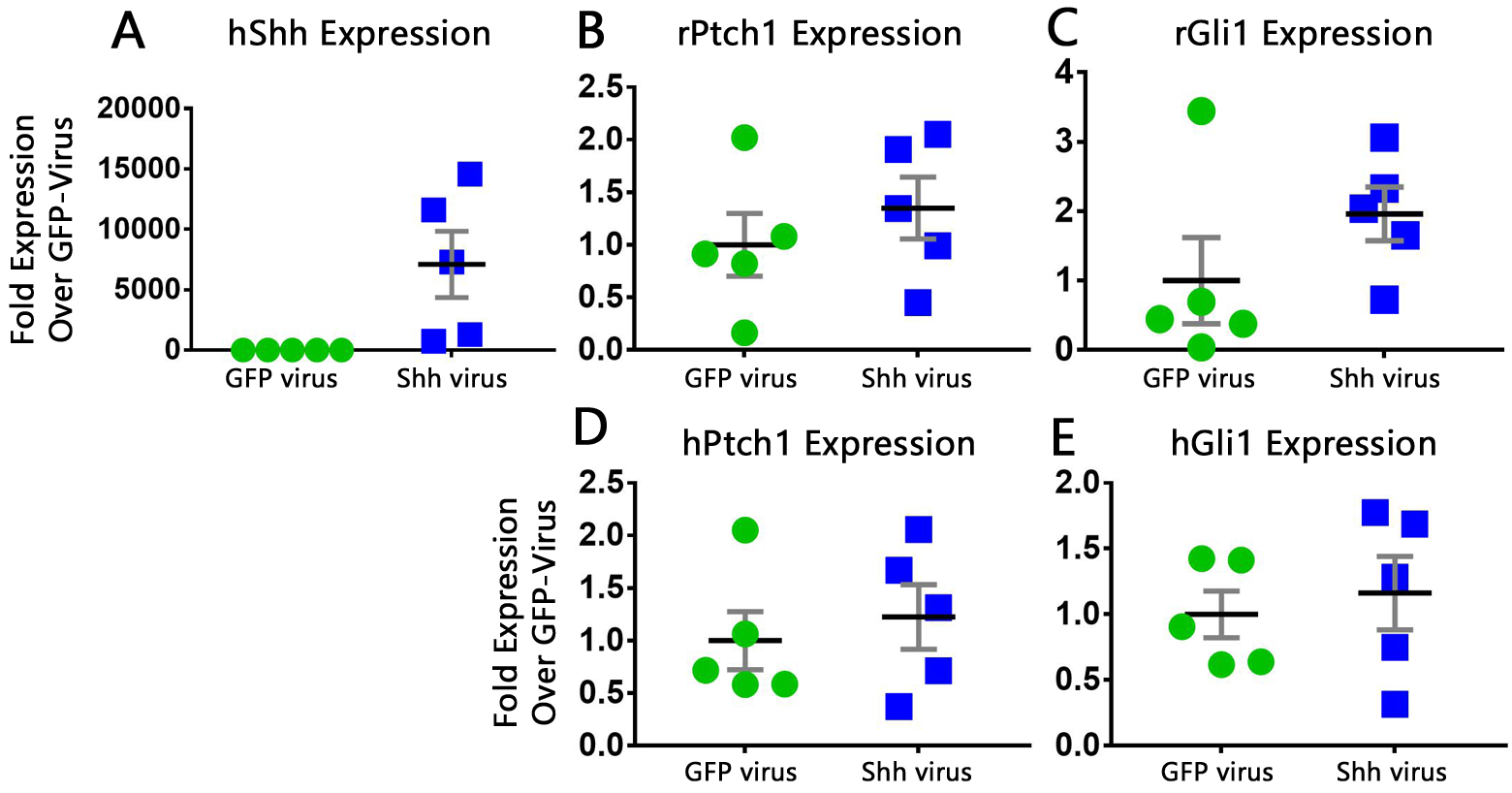

Supplement: S1 Fig — qRT-PCR results from infarcted and cell engrafted animals that received a 109 vp dose of either the GFP virus or the hShh virus. RNA was isolated from tissue that had been fixed and paraffin embedded, and therefore reflects levels from within the entire heart, not the localized areas as in Fig 1. As such, these results show lower and more inconsistent upregulation. (A) hShh RNA levels were increased in all animals that received the Shh virus. (B,C) Upregulation of rPtch1 (B) and rGli1 (C) did not reach significance, but did show strong trends toward increased expression. (D,E) We also determined if hSC-CMs responded to Shh by upregulating the human forms of the downstream Hh pathway components Ptch1 and Gli1. The injected hSC-CMs do not show a measurable increase in the amounts of hPtch1 (D) or hGli1 (E) RNA as compared to those treated with GFP virus. Therefore, the engrafted cells do not contribute to the spread of pathway activation within the scar. (TIF) [file pone.0227780.s002.tif]

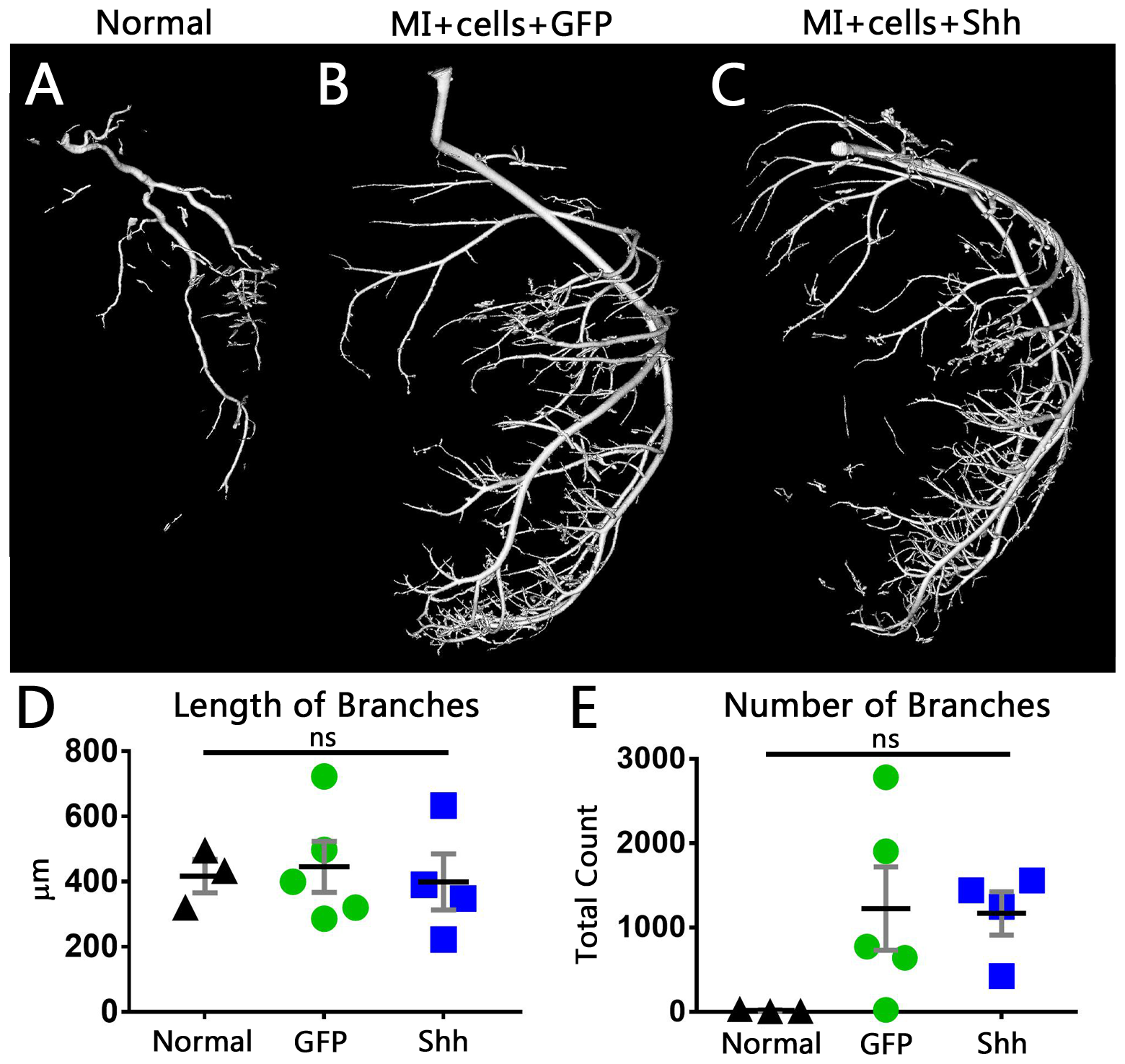

Supplement: S2 Fig — (A-C) 3D reconstructions of the RCA within normal hearts (A) or hearts that were infarcted, and injected with hSC-CMS after treatment with either GFP virus (B) or Shh virus (C). (D,E) Quantification of the branching structure. Black line is mean, error bars are SEM. (TIF) [file pone.0227780.s003.tif]

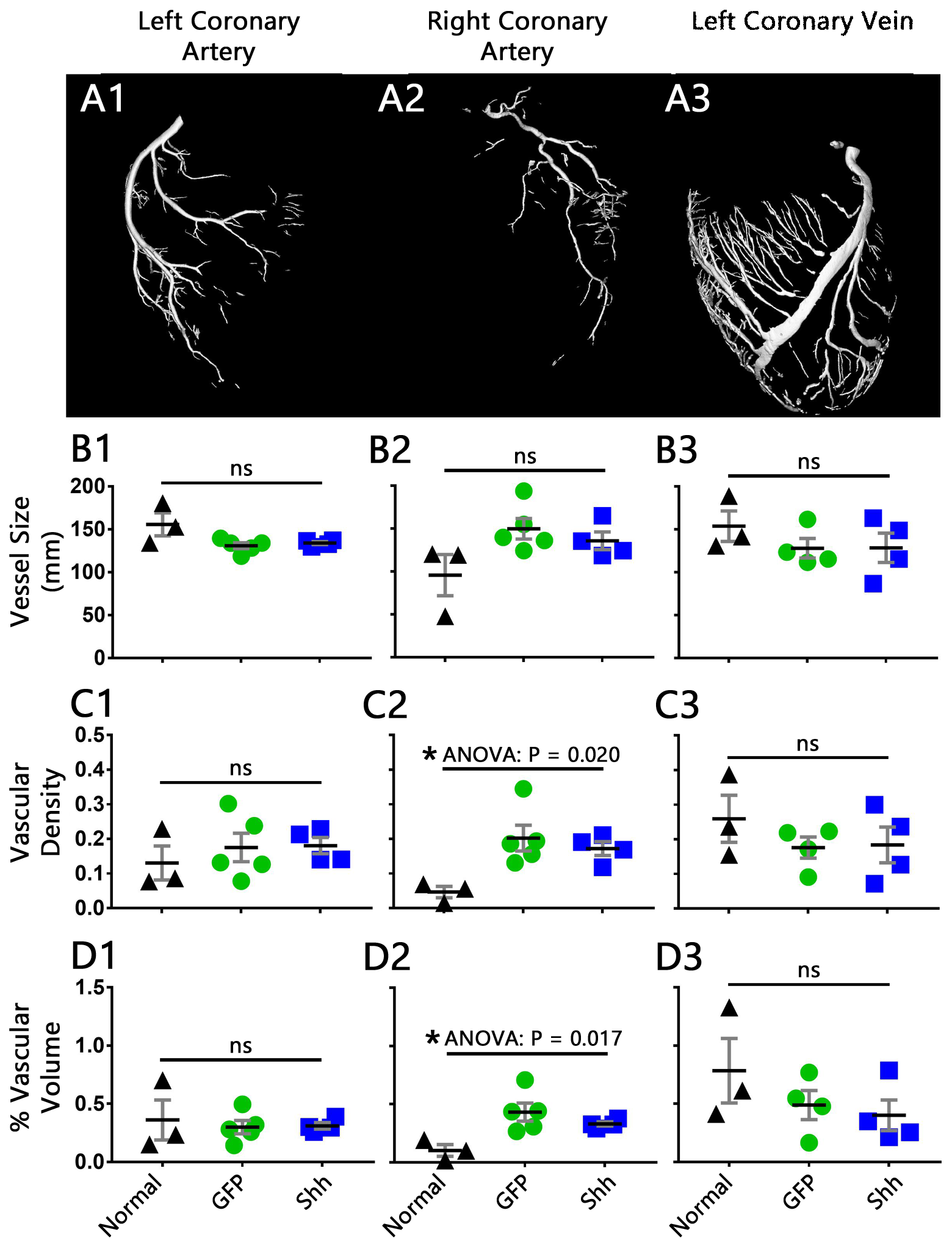

Supplement: S3 Fig — (A1–3) 3D reconstructions of the vasculature within a normal heart, segmented to show the vascular subset analyzed within each column: LCA (column 1), RCA (column 2), LCV (column 3). (B-D) Quantification of vascular measurements, as specified. Vessel size is equivalent circular diameter (2D vessel cross sections, B), vascular density is number of vessels per square mm (2D cross sections, C), and percent vascular volume is the volume of vessels compared to the volume of heart tissue (calculations in 3D, D). Black line is mean, error bars are SEM. Reported P values are from the ANOVA analysis, not the Tukey’s post-test. (TIF) [file pone.0227780.s004.tif]
